# Disparities in attendance at diabetes self-management education programs after diagnosis in Ontario, Canada: a cohort study

**DOI:** 10.1186/1471-2458-13-85

**Published:** 2013-01-30

**Authors:** Karen Cauch-Dudek, J Charles Victor, Marianne Sigmond, Baiju R Shah

**Affiliations:** 1 Institute for Clinical Evaluative Sciences, G106 – 2075 Bayview Avenue, Toronto, ON, M4N 3M5, Canada; 2 Dalla Lana School of Public Health, University of Toronto, Toronto, Canada; 3 Division of Endocrinology, University Health Network, Toronto, Canada; 4 Department of Medicine, University of Toronto, Toronto, Canada; 5 Department of Medicine, Sunnybrook Health Sciences Centre, Toronto, Canada

**Keywords:** Diabetes self-management education programs, Utilization of health services, Disparities, Population-based research

## Abstract

**Background:**

Patients newly-diagnosed with diabetes require self-management education to help them understand and manage the disease. The goals of the study were to determine the frequency of diabetes self-management education program utilization by newly-diagnosed patients, and to evaluate whether there were any demographic or clinical disparities in utilization.

**Methods:**

Using population-level health care data, all 46,553 adults who were diagnosed with any type of non-gestational diabetes in Ontario, Canada between January and June 2006 were identified. They were linked with a diabetes self-management education program registry to identify those who attended within 6 months of diagnosis. The demographic and clinical characteristics of attendees and non-attendees were compared.

**Results:**

A total of 9,568 (20.6%) patients attended a diabetes self-management education program within 6 months of diagnosis. Younger age, increasing socioeconomic status, and the absence of mental health conditions or other medical comorbidity were associated with attendance. Patients living in rural areas, where access to physicians may be limited, were markedly more likely to attend. Recent immigrants were 40% less likely to attend self-management education programs than longer-term immigrants or nonimmigrants.

**Conclusion:**

Only one in five newly-diagnosed diabetes patients attended a diabetes self-management education program. Demographic and clinical disparities in utilization persisted despite a publicly-funded health care system where patients could access these services without direct charges. Primary care providers and education programs must ensure that more newly-diagnosed diabetes patients receive self-management education, particularly those who are older, poorer, sicker, or recent immigrants.

## Background

Diabetes mellitus is a common and serious chronic disease, and is associated with impaired quality of life, premature mortality and significant economic costs [1-3]. Effective management of diabetes can reduce the risk of complications and morbidity for people with diabetes, but these approaches are complex as they require sustained efforts at behavior modification and long-term preventative pharmacological therapy [4]. Thus, many jurisdictions have adopted a chronic disease management model centered on patient self-management supported by multidisciplinary health care teams to help address this epidemic [5-7]. Comprehensive diabetes self-care education is necessary to successfully implement and sustain lifestyle changes, and to ensure that patients are utilizing appropriate health services. Reviews of randomized trials have suggested that self-management education can lead to improvements in knowledge, dietary habits, frequency and accuracy of glucose self-monitoring, weight and glycemic control [8-16]. It has also been found to be cost-effective, or even cost-saving [17-19]. Arguably the most important time for patients to receive self-management education is immediately after diagnosis. At that time, patients are seeking information about their new condition, and they may be overwhelmed by the many behavioral changes they are asked to adopt almost immediately [20]. Thus, the time immediately following diagnosis represents a critical window when patients are most likely to be interested in chronic disease care and receptive to self-management education.

However, as for other health care services, there may be disparities in utilization of self-management education across different populations. A number of small American studies have used cross-sectional self-reported survey data to show that certain patient subgroups, such as those with lower education or older age, are less likely to report having previously received diabetes education [21-23]. However, in addition to the methodological limitations of the design and data sources of these studies, their results may not be generalizable to health care systems offering publicly-funded universal access for all patients, where many disparities in care might be mitigated. Thus, the purposes of this population-based cohort study were to determine the frequency of diabetes self-management program utilization by patients within 6 months of diagnosis in Ontario, Canada, and to evaluate whether there were any demographic or clinical disparities in utilization.

## Methods

This population-based cohort study used health care administrative databases that detail health care utilization by every resident of Ontario, Canada’s most populous province with over 12 million residents. These data sources included: 1) the Registered Persons Database, a register of demographic information about all Ontario residents; 2) the physician service claims database, which records all fee-for-service billing claims from Ontario physicians; 3) the discharge abstracts database, which records detailed information about each hospitalization to an Ontario hospital; and 4) the Ontario Diabetes Database, a validated registry of all people diagnosed with non-gestational diabetes in Ontario, which is derived from these three administrative databases [24]. The registry was developed by and is updated annually by the Institute for Clinical Evaluative Sciences, and uses the diagnosis codes recorded on physician service claims and hospitalization records to identify cases of diabetes. The Ontario Diabetes Database does not distinguish between type 1, type 2 or other diabetes, so the analyses could not be divided according to diabetes type. Individuals are linked between all of these databases using their unique health card number, which is used by all Ontario residents to access health care services.

A cohort was defined of every adult aged ≥18 years in Ontario who was diagnosed with diabetes between January and June 2006, according to the Ontario Diabetes Database. Patients who had immigrated within 2 years prior to the diagnosis of diabetes were excluded, as their apparently incident diabetes in the registry might have represented prevalent diabetes at the time of immigration. Baseline demographic and clinical characteristics that might be associated with disparities in utilization of self-management education programs after diabetes diagnosis were defined using the linked administrative data. These included: age, sex, socioeconomic status (derived ecologically as the median neighborhood household income divided into quintiles), immigration within the previous 10 years, rural residence, primary care visits in the year prior to diabetes diagnosis, diabetes diagnosed during a hospitalization, previous cardiac events (hospitalizations for myocardial infarction, angina or coronary bypass within 5 years prior to diabetes diagnosis), previous mental illness (based on a validated method using physician service claims) [25], and medical comorbidity (score of ≥1 on the Charlson comorbidity index) [26].

Most diabetes self-management education in Ontario is delivered through 331 diabetes self-management education programs distributed throughout the province. These programs are funded in whole or in part by the Ministry of Health, and most are hosted by academic or community hospitals, community health centres or First Nations organizations. There was no existing province-wide registry or administrative database with information on diabetes self-management education program visits. Therefore, a registry was compiled by collecting the visit dates and health card numbers of all adults attending any diabetes self-management education program in Ontario during the 2006 calendar year. Data were collected either through data extraction from existing electronic medical records or manual chart abstractions conducted by program staff or trained chart abstractors. These data were then linked to the other population-level health care data through the patients’ health card numbers. The primary outcome was a visit to any diabetes self-management education program within 6 months of diabetes diagnosis. This follow-up period after diabetes diagnosis was selected as a reasonable time in which patient self-management education should be implemented [6,7]. Patients who died prior to the end of follow-up were excluded.

To determine whether wait times for diabetes self-management education might contribute to low utilization, a subgroup analysis was performed expanding the follow-up window to 8 months after diabetes diagnosis, among the subgroup of patients who had sufficient follow-up time (i.e., those who were diagnosed with diabetes between January and April 2006).

Bivariate associations between demographic and clinical characteristics and diabetes self-management education program attendance were examined using chi-square tests of association. To examine the independent effects of these characteristics on program attendance, a multivariable log-binomial regression model was used to estimate adjusted relative risks. All analyses were performed using SAS version 9.2 (Cary, NC).

This study was approved by the research ethics board of Sunnybrook Health Sciences Centre. Local ethics approval was also obtained from 48 additional ethics boards, medical advisory boards or ethics consultations, as requested by individual programs during the course of registry compilation.

## Results

There were 50,363 adults newly diagnosed with diabetes in Ontario between January and June 2006. Of them, 1,205 were excluded because they died before December 31, 128 were excluded because of missing address information needed to determine baseline demographic characteristics, and 2,477 were excluded because, as recent immigrants to Ontario, we could not distinguish incident from prevalent diabetes. Of the final cohort of 46,553 people, only 9,568 (20.6%) attended a diabetes self-management education program within 6 months of their diabetes diagnosis. In the subgroup of 31,580 patients who were diagnosed with diabetes between January and April 2006, 20.6% had a program visit within 6 months, growing only to 22.0% when follow-up was lengthened to 8 months.

Unadjusted and fully adjusted associations between the baseline characteristics and diabetes self-management education program attendance are shown in Table 1. Increasing age was associated with decreased attendance in both the unadjusted analysis and in the adjusted regression model, with only 11.9% of patients aged ≥80 at diagnosis attending a diabetes self-management education program. In contrast, increasing socioeconomic status was associated with increased attendance. There were no significant differences in program attendance between men and women. The largest absolute difference in attendance was between patients residing in rural regions versus those in non-rural regions, with nearly one-third of rural residents attending a diabetes self-management education program. Recent immigrants were markedly less likely to attend a program. Patients with no family physician visits in the preceding year and those with seven or more visits were less likely to attend a diabetes self-management education program than those with one to six visits in the preceding year. Finally, patients with mental health conditions or medical comorbidity were somewhat less likely to attend.

**Table 1:** Associations between the baseline demographic and clinical characteristics, and diabetes self-management education program utilization

		**Diabetes self-management education program attendees**	**Diabetes self-management education program non-attendees**	**Unadjusted p-value**	**Adjusted relative risk**	**Adjusted p-value**
		**(n = 9,899) %**	**(n = 39,131) %**		**(95% CI)**	
Age	18-44	1,884 (19.7)	6,564 (17.7)	<0.001	1.00	<Ref
	45-54	2,330 (24.4)	8,008 (21.7)		0.96 (0.91–1.02)	0.177
	55–64	2,599 (27.2)	9,420 (25.5)		0.89 (0.85–0.94)	<0.001
	65-79	2,362 (24.7)	10,085 (27.3)		0.79 (0.75–0.84)	<0.001
	80+	393 (4.1)	2,908 (7.9)		0.52 (0.47–0.57)	<0.001
Female sex		4,453 (46.5)	17,296 (46.8)	0.695	1.03 (1.00–1.07)	0.078
Socioeconomic status	Lowest	1,956 (20.4)	8,379 (22.7)	<0.001	1.00	<Ref
	2	1,940 (20.3)	8,194 (22.2)		1.01 (0.95–1.07)	0.757
	3	1,982 (20.7)	7,434 (20.1)		1.08 (1.03–1.15)	0.003
	4	1,928 (20.2)	6,912 (18.7)		1.11 (1.05–1.18)	<0.001
	Highest	1,762 (18.4)	6,066 (16.4)		1.13 (1.07–1.20)	<0.001
Recent immigrant		541 (5.7)	3,658 (9.9)	<0.001	0.60 (0.55–0.65)	<0.001
Rural residence		1,692 (17.7)	3,684 (10.0)	<0.001	1.59 (1.52–1.66)	<0.001
Primary care visits in year prior to diagnosis	0	627 (6.6)	2,624 (7.1)	<0.001	0.87 (0.81–0.94)	<0.001
	1–6	5,998 (62.7)	21,397 (57.9)		1.00	<Ref
	7–12	2,189 (22.9)	8,943 (24.2)		0.96 (0.91–1.00)	0.047
	13+	754 (7.9)	4,021 (10.9)		0.79 (0.74–0.85)	<0.001
Diabetes diagnosed during a hospitalization		591 (6.2)	2,726 (7.4)	<0.001	0.96 (0.88–1.04)	0.301
Previous cardiac event		367 (3.8)	1,659 (4.5)	0.005	0.95 (0.86–1.04)	0.272
Mental health condition		2,805 (29.3)	11,548 (31.2)	<0.001	0.94 (0.91–0.98)	0.006
Medical comorbidity		499 (5.2)	2,592 (7.0)	<0.001	0.89 (0.81–0.97)	0.010

## Discussion

Only one in five adults with newly-diagnosed diabetes in Ontario attended a diabetes self-management education program within 6 months of diagnosis. The remaining patients therefore either received no formal professional self-management education about their new condition, or they received education during other health care encounters such as visits with their family physician, even though the education that can be provided in this context is often inadequate, in part because primary care physicians often misunderstand patients’ informational needs at diagnosis [20]. This low utilization of diabetes self-management education programs occurred despite the fact that these services are available without direct patient charges and often without requiring physician referral, and despite the fact that these services are broadly distributed throughout the province, reducing geographic barriers to access.

Younger patients were more likely to attend a diabetes self-management education program than older patients. This may be due in part to a higher likelihood of type 1 diabetes among young adults with newly diagnosed diabetes. However, the gradient of decreasing utilization with increasing age persisted across all age strata, such that those aged over 80 years were nearly half as likely to attend a self-management education program as young adults. Like advancing age, people with lower socioeconomic status, those who were recent immigrants, and those with mental health conditions or medical comorbidity were less likely to attend a diabetes self-management education program, despite arguably being more in need of self-management support. The observed results are consistent with previous studies, where utilization was found to be lower amongst older adults and those with lower levels of education [22,23,27]. A more recent survey of 295 patients with diabetes in Pennsylvania found that only 45% reported having ever received self-management education, and patients with complications or with poor control were no more likely to have attended [28]. A Canadian survey of 781 patients found that demographic factors did not predict utilization of diabetes education [29]. Surveys of health care providers in the United States and Canada have suggested that barriers to utilization include patient unwillingness, programs’ location, languages of service, operating hours, and (in the United States) insurance coverage [30-32]. The disparities in utilization found in this study may also partially explain the observation that patients utilizing education services had lower average costs than non-attendees [17], since older, poorer and sicker patients who would be predicted to have higher health care costs were also less likely to use such services in the first place. Nonetheless, the presence of these disparities in diabetes self-management education program utilization is noteworthy in a publicly-funded health care system where patients are meant to have equitable access to these services. These populations may face other barriers to access and utilization, such as lower health literacy, less ability to navigate the health care system, or occupational or financial barriers to attending programs.

Patients living in rural areas were markedly more likely to attend diabetes self-management education programs, perhaps because other providers of diabetes care, such as family physicians or endocrinologists, are more difficult to access in rural areas. We had hypothesized that patients who had no family physician visits prior to diabetes diagnosis might have had greater utilization of diabetes self-management education programs as an alternative source of primary diabetes care. Instead, these patients were less likely to have program utilization. Interestingly, patients with many family physician visits prior to diagnosis were also less likely to attend, perhaps because such frequent visits are a marker for other comorbidity or high demand health needs that distract from optimal chronic disease management. We had also hypothesized that patients whose diabetes was diagnosed during a hospitalization might be less likely to attend a diabetes self-management education program, reflecting inadequate attention to chronic disease follow-up upon discharge from hospital. However, attendance did not differ for these patients.

One possible reason for low utilization within the first 6 months after diagnosis could be that wait times for receiving diabetes self-management education services exceeded 6 months. If this were the case, we would expect that examining longer periods of follow-up would lead to ongoing increases in the proportion of patients who attended diabetes education. However, when we reanalyzed the data by lengthening follow-up by one-third to 8 months, the proportion of patients attending a diabetes self-management education program increased by only 1.4%. Hence, it is unlikely that wait times were a significant contributor to low utilization.

This study is unique in a number of ways. It is the first study to examine demographic and clinical predictors of diabetes self-management education program utilization in Canada’s universal health care system, where socioeconomic status and insurance are not the overwhelming barriers to health care utilization that they are in other health care systems. The study shows that even in this universal access context, disparities by age, socioeconomic status and immigration history occur. Virtually all previous studies of diabetes self-management education utilization have relied on small cross-sectional surveys of patients with diabetes; our study examined real-world utilization of self-management education services at a population level in a longitudinal cohort, making it more methodologically rigorous. However, there are some limitations to note. First, although our cohort of patients was linked to large health care databases permitting measurement of many important demographic and clinical predictor variables, other potentially important factors that might be associated with disparities in diabetes self-management education program attendance could not be measured from population-level data, such as education level, employment, health literacy or language proficiency. Second, as noted above, the Ontario Diabetes Database does not distinguish between types of diabetes, so we could not specifically compare predictors of program attendance between type 1 and type 2 diabetes. Third, there are undoubtedly many patients who were referred to self-management education programs who did not ultimately attend. Since our study only captured attendance, these patients would not have been identified. However, our study focused on the outcome measure of program attendance, not on the process measure of receiving a referral to one from a health care provider. Fourth, the influence of variability in the curricula between diabetes education programs could not be evaluated in our study, as these data were not available. However, a Standards Recognition Program for diabetes education has been developed by the Canadian Diabetes Association, so the extent of variability may not be significant. In addition, each program receives at least partial funding from the provincial Ministry of Health, and therefore must adhere to government-mandated standards on curriculum, staffing and access. Finally, our study examined attendance only at formal diabetes self-management education programs. Although these programs are the main sources for self-management education, other sources of self-management education and support (such as an individual nurse working with a primary care physician, or self-management education provided by a pharmacist in a dispensary) could not be captured by this study.

Further research is required to determine what patient, provider and health care system factors contribute to the observed disparity in utilization by vulnerable patients. In addition, interventions to improve utilization of self-management education programs by patients newly-diagnosed with diabetes need to be developed and evaluated.

## Conclusions

In summary, patients with diabetes express a strong preference to receive self-management education soon after diagnosis, as they have many immediate issues about management that need to be addressed [20]. In a randomized trial, attending a self-management education program at diagnosis led to improved understanding of the illness, and these changes in illness beliefs were correlated with metabolic changes [33]. However, only one in five people with newly diagnosed diabetes in the publicly-funded health care system of Ontario attended a diabetes self-management education program. Utilization was lower in the population subgroups most in need of self-management support: older, poorer individuals, recent immigrants, and those with mental health conditions or medical comorbidities. Clinicians managing patients with diabetes are doing an inadequate job ensuring that their patients newly-diagnosed with the condition are receiving diabetes self-management education at a time when they are seeking information about the disease [20]. Strategies such as increasing primary care provider awareness of self-management education program services, or facilitating and coordinating referral processes, may increase utilization rates. In the subgroups of the population where disparities in utilization are seen, particular focus is needed to ensure these patients have equitable access to services. For example, placing self-management education programs in poorer neighborhoods or offering evening or weekend opening hours may address disparities for lower income patients who could not otherwise access these programs. Multilingual culturally sensitive programming may improve utilization by recent immigrants. Such strategies are needed to help these disadvantaged patients, who are at increased risk for diabetes complications, to optimize their care.

## Competing interests

The authors declare that they have no competing interests.

## Authors’ contributions

KCD contributed to the conception and design of the study, the acquisition and interpretation of data, and the drafting of the manuscript. JCV contributed to the interpretation of data. MS contributed to the conception and design of the study and the interpretation of data. BRS contributed to the conception and design of the study, and the acquisition and interpretation of data. All authors contributed to the revision of the manuscript for important intellectual content, and read and approved the final manuscript.

## Pre-publication history

The pre-publication history for this paper can be accessed here:

http://www.biomedcentral.com/1471-2458/13/85/prepub

## References

[B1] Stewart AL Greenfield S Hays RD Wells K Rogers WH Berry SD Functional status and well-being of patients with chronic conditions. Results from the Medical Outcomes Study JAMA 1989 262 907 913 10.1001/jama.1989.03430070055030 2754790

[B2] Gregg EW Gu Q Cheng YJ Narayan KMV Cowie CC Mortality trends in men and women with diabetes, 1971 to 2000 Ann Intern Med 2007 147 149 155 17576993 10.7326/0003-4819-147-3-200708070-00167

[B3] Dawson KG Gomes D Gerstein H Blanchard JF Kahler KH The economic cost of diabetes in Canada, 1998 Diabetes Care 2002 25 1303 1307 10.2337/diacare.25.8.1303 12145225

[B4] Bodenheimer T Wagner EH Grumbach K Improving primary care for patients with chronic illness: the chronic care model, part 2 JAMA 2002 288 1909 1914 10.1001/jama.288.15.1909 12377092

[B5] International Diabetes Federation Clinical Guidelines Taskforce Global Guideline For Type 2 Diabetes 2005 Brussels: International Diabetes Federation

[B6] American Diabetes Association Standards of medical care in diabetes − 2012 Diabetes Care 2012 34 S11 S63 10.2337/dc12-s011 PMC3632172 22187469

[B7] Canadian Diabetes Association Clinical Practice Guidelines Expert Committee Canadian Diabetes Association 2008 clinical practice guidelines for the prevention and management of diabetes in Canada Can J Diab 2008 32 S1 S201 10.1016/j.jcjd.2013.01.009 24070926

[B8] Duke S-AS Colagiuri S Colagiuri R Individual patient education for people with type 2 diabetes mellitus Cochrane Database Syst Rev 2009 1 CD005268 19160249 10.1002/14651858.CD005268.pub2 PMC6486318

[B9] Loveman E Frampton GK Clegg AG The clinical effectiveness of diabetes education models for type 2 diabetes: a systematic review Health Technol Assess 2008 12 1 116 10.3310/hta12090 18405469

[B10] Deakin T McShane CE Cade JE Williams RDRR Group based training for self-management strategies in people with type 2 diabetes mellitus Cochrane Database Syst Rev 2005 2 CD003417 15846663 10.1002/14651858.CD003417.pub2

[B11] Ellis SE Speroff T Dittus RS Brown A Pichert JW Elasy TA Diabetes patient education: a meta-analysis and meta-regression Patient Educ Couns 2004 52 97 105 10.1016/S0738-3991(03)00016-8 14729296

[B12] Warsi A Wang PS LaValley MP Avorn J Solomon DH Self-management education programs in chronic disease: a systematic review and methodological critique of the literature Arch Intern Med 2004 164 1641 1649 10.1001/archinte.164.15.1641 15302634

[B13] Gary TL Genkinger JM Guallar E Peyrot M Brancati FL Meta-analysis of randomized educational and behavioral interventions in type 2 diabetes Diabetes Educ 2003 29 488 501 10.1177/014572170302900313 12854339

[B14] Loveman E Cave C Green C Royle P Dunn N Waugh N The clinical and cost-effectiveness of patient education models for diabetes: a systematic review and economic evaluation Health Technol Assess 2003 7 22 1 190 10.3310/hta7220 13678547

[B15] Norris SL Lau J Smith SJ Schmid CH Engelgau MM Self-management education for adults with type 2 diabetes: a meta-analysis of the effect on glycemic control Diabetes Care 2002 25 1159 1171 10.2337/diacare.25.7.1159 12087014

[B16] Norris SL Engelgau MM Narayan KMV Effectiveness of self-management training in type 2 diabetes: A systematic review of randomized controlled trials Diabetes Care 2001 24 561 587 10.2337/diacare.24.3.561 11289485

[B17] Duncan I Birkmeyer C Coughlin S Li Q Sherr D Boren S Assessing the value of diabetes education Diabetes Educ 2009 35 752 760 10.1177/0145721709343609 19783766

[B18] Boren SA Fitzner KA Panhalkar PS Specker JE Costs and benefits associated with diabetes education Diabetes Educ 2009 35 72 96 10.1177/0145721708326774 19244564

[B19] Urbanski P Wolf A Herman WH Cost-effectiveness of diabetes education J Am Diet Assoc 2008 108 S6 S11 18358259 10.1016/j.jada.2008.01.019

[B20] Beeney LJ Bakry AA Dunn SM Patient psychological and information needs when the diagnosis is diabetes Patient Educ Couns 1996 29 109 116 10.1016/0738-3991(96)00939-1 9006227

[B21] Coonrod BA Betschart J Harris MI Frequency and determinants of diabetes patient education among adults in the U.S. population Diabetes Care 1994 17 852 858 10.2337/diacare.17.8.852 7956630

[B22] Graziani C Rosenthal MP Diamond JJ Diabetes education program use and patient-perceived barriers to attendance Family Med 1999 31 358 363 10407715

[B23] Glasgow RE Toobert DJ Hampson SE Participation in outpatient diabetes education programs: how many patients take part and how representative are they? Diabetes Educ 1991 17 376 380 10.1177/014572179101700509 1879279

[B24] Hux JE Ivis F Flintoft V Bica A Diabetes in Ontario: determination of prevalence and incidence using a validated administrative data algorithm Diabetes Care 2002 25 512 516 10.2337/diacare.25.3.512 11874939

[B25] Steele LS Glazier RH Lin E Evans M Using administrative data to measure ambulatory mental health service provision in primary care Med Care 2004 42 960 965 10.1097/00005650-200410000-00004 15377928

[B26] Charlson ME Pompei P Ales KL MacKenzie CR A new method of classifying prognostic comorbidity in longitudinal studies: Development and validation J Chron Dis 1987 40 373 383 10.1016/0021-9681(87)90171-8 3558716

[B27] Forjuoh SN Huber C Bolin JN Patil SP Gupta M Helduser JW Provision of counseling on diabetes self-management: Are there any age disparities? Patient Educ Couns 2011 85 133 139 10.1016/j.pec.2010.08.004 20863646 PMC3021766

[B28] Ruppert K Uhler A Siminerio L Examining patient risk factors, comorbid conditions, participation, and physician referrals to a rural diabetes self-management education program Diabetes Educ 2010 36 603 612 10.1177/0145721710369705 20479132

[B29] Shah BR Booth GL Predictors and effectiveness of diabetes self-management education in clinical practice Patient Educ Couns 2009 74 19 22 10.1016/j.pec.2008.08.005 18805668

[B30] Peyrot M Rubin RR Access to diabetes self-management education Diabetes Educ 2008 34 90 97 10.1177/0145721707312399 18267995

[B31] Gucciardi E Chan VW Fortugno M Khan S Horodezny S Swartzack SS Primary care physician referral patterns to diabetes education programs in southern Ontario, Canada Can J Diabetes 2011 35 26 268

[B32] Shaw K Killeen M Sullvian E Bowman P Disparities in diabetes self-management education for uninsured and underinsured adults Diabetes Educ 2011 37 813 819 10.1177/0145721711424618 22021026

[B33] Davies MJ Heller S Skinner TC Campbell MJ Carey ME Cradock S Effectiveness of the diabetes education and self management for ongoing and newly diagnosed (DESMOND) programme for people with newly diagnosed type 2 diabetes: cluster randomised controlled trial BMJ 2008 336 491 495 10.1136/bmj.39474.922025.BE 18276664 PMC2258400

